# Dr. Mossman, on Consumption

**Published:** 1800-10

**Authors:** Geo. Mossman

**Affiliations:** Bradford


### *To the Editors of the Medical and Physical Journal.*

GENTLEMEN,

MY prediction, respecting the future reputation of the *Fox-glove*, will, I have no doubt, be eventually accomplished. In this town and neighbourhood the plant is now in more general use than any other healing agent; and either in a solitary or in a combined form, it is employed in almost every case of *increased vascular action.*

In *pneumonic inflammation*, and in *active hæmorrhagies*, it certainly possesses powers approximating to specific; and even in cases of *continued fever*, of various types, I have repeatedly witnessed the most beneficial effects from its administration.

This

This high character of the plant has not been acquired by the exertion of any authority in its favour, but by the ſtriking proofs which it continues to diſplay of its curative powers. In oppoſition to much preconceived opinion, and much ſtrong prejudice, I know that it has forced itſelf upon the notice of many of my friends, and now forms a very principal inſtrument of their practice. This general employment of the *Digitalis* has excited much attention to the collecting of the leaves, and to the mode of preparing them for uſe; and I imagine that the Medical Gentlemen of this place, keep the plant in as high a ſtate of perfection as it can be met with in any part of the kingdom.

I have obſerved, in another place, that the proper doſe of a medicine is juſt that quantity which will produce the deſired effect; and that the proper doſe of the *Digitalis* is that which, by gradually bringing the ſyſtem under its influence, will ſo regulate the motion of the heart, as to reduce the pulſations of the artery to a given number.

I think that the choice of the preparation to be employed may be left to the diſcretion of the preſcriber; for if his principal object be the diminution of vaſcular action, I know that he can very certainly accompliſh this end, either by means of the *powder* or the *tincture* of the plant; any diſputation, therefore, about which of thoſe two we ought to employ, has always appeared to me unneceſſary.

For upwards of twelve months I have preſcribed the *Digitalis* very extenſively; and during the laſt ſix of that period I have had very ample experience of its powers. I am now fully perſuaded, that, by a judicious management of the plant variouſly combined, I can obviate *pneumonic inflammation with as much certainty as I can arrest the progress of an intermittent fever by means of the bark of the cinchona.* Again, I am perſuaded that, if *pulmonary consumption be divided into four stages, the Digitalis will very certainly cure the three first, and as certainly alleviate the distressing symptoms of the last.*

I am aware that the introduction of a new remedy is a taſk difficult and dangerous; for I am aware that there are men, who will not only oppoſe, but cenſure with much aſperity, the application of any healing power which has originated with, or been patronized by, a rival practitioner. This narrow principle is the reproach of individuals, and the diſgrace of ſcience, Some time ago, in conſultation, I recommended a trial of the *Digitalis* in a caſe of *phthisis.* It was contended that the plan would avail nothing; nay, that its effects would be highly injurious. I aſked my medical friend, if he had ever employed the *Fox-glove* in caſes of *pulmonary consumption?* "I have not" was his reply. "Which of us, then," I rejoined, "is beſt entitled to decide on its effects; you, who have never exhibited it at all; or I, who have adminiſtered it a hundred times?

In my eſtimation, a man becomes truly contemptible when he either betrays a haughty impatience of every improvement upon ſubjects of ſpeculation, or when, by an ungenerous exertion of overgrown authority, he is captiouſly ſolicitous to doom to diſregard every opinion which is not his own. It is certainly not unreaſonable to demand that the *Digitalis* ſhould have the ſame fair trial which other remedies have had; and that it ſhould *be exhibited in a genuine form, in the doses, and in those diseases, for which it has been recommended by its advocates.*— The ſubſtantial qualities of the plant will thus be very certainly aſcertained. It is admitted on all hands, that the *Digitalis* is a plant of much activity; it ſurely, then, becomes a matter of high intereſt to know, whether its powers are uniformly and poſitively deſtructive; or, whether it be poſſible to apply them to ends the moſt uſeful and ſalutary. If its powers be found to admit of a beneficial application, let them command the protection of men of ſcience: if they be injurious, let them be doomed to diſuſe by the ſame authority. Let not the diſcuſſion of the queſtion be embarraſſed by narrow jealouſies, but let it aſſume an open complexion, diſtinguiſhed by accuracy of experiment and cloſeneſs of obſervation. In diſquiſitions which embrace a large portion of the deſtiny of man, it is pitiful to ſee talents employed in peeviſhly frittering away the attainments of their cotemporaries, rather than in a manly and diſpaſſionate purſuit of uſeful knowledge. I venerate every improvement, the tendency of which is to ameliorate the condition of us all; but I beg leave to expreſs my aſtoniſhment, that the ſubjects of *Vaccine Inoculation*, and the *antisyphilitic virtues of the Nitric Acid*, ſhould engroſs the attention of almoſt all the medical part of Europe; and that the inquiry reſpecting the curative agency of the *Digitalis* ſhould ſtill be confined to a few names and to a few places. Since the introduction of the *variolous contagion* by means of *inoculation*, very few, comparatively, have periſhed by that diſeaſe; ſince the period that the *antisyphilitic* powers of *mercury* were generally acknowledged, a very ſmall proportion of thoſe labouring under *venereal complaints* have fallen victims to their malignity: But it is ſaid, that *eighty thousand* of the inhabitants of this iſland periſh annually by *pulmonary consumption*; and yet an inveſtigation of the means by which this gigantic evil may be obviated, excites only a very partial intereſt among the profeſſors of the healing art.

I tranſmit

I tranſmit to you the following additional caſes, in which the *Digitalis* was ſuccefsfully employed; and am,

Gentlemen,

Your's, obediently,

*Bradford, Aug.* 15, 1800. GEO. MOSSMAN.

---

Case XII.—Sept. 24, 1799, N. H. æt. 19, for ſix months previous to this date has laboured under *phthisical symptoms*, accompanied by a conſiderable *enlargement of the abdomen.* She complains of a teazing cough, difficulty of breathing, alternate chills and heats, great thirſt, loſs of appetite, ſtrength and fleſh. Her abdomen is much tumefied, and ſhe experiences much ſorenefs from preſſure; her urine has very generally been high coloured, and her complaints have been all along attended by an occaſional irregularity in the ſtate of her bowels; her pulſe is 120. She has had a reſpectable medical attendant, and by him ſhe has been treated with ſmall doſes of the *Fox-glove*, and ſome preparation of *steel*; from which, however, ſhe thinks ſhe has not derived any conſiderable advantage. I adviſed her diet to conſiſt principally of preparations of *milk*, *eggs*, and *animal food*; I alſo directed *frictions* to be applied to the region of the abdomen; and ordered for her the ſubſequent formula:

℞. Calomel gr. xv. Fol. Digital. *recenter pulverizat.* ʒj. Miſce et adde Conſerv. Cynoſbat. q. s. ut fiant pilulæ lx.—Sumat j. quater in die.

Sept. 27th. She is now obviouſly under the influence of the *Digitalis*, more in conſequence, I apprehend, of what ſhe took previous to my ſeeing her, than from the quantity of the plant which ſhe has taken ſince. She complains much of giddineſs, and a dimneſs of viſion; ſhe experiences a faintneſs at her ſtomach, approximating to a ſlight nauſea; her pulſe is 58, with a little intermiſſion; her cough is better; and ſhe thinks that ſhe is ſomewhat diminiſhed in ſize. The rigors and ſucceeding heats, ſhe ſays, have left her; and her thirſt is conſiderably abated.

Oct. 2d. Yeſterday morning ſhe was ſuddenly ſeized with vomiting, which continued for the whole of the day, and abated towards the evening, without her having recourſe to any antidote. Her pulſe is 50, with very conſiderable intermiſſion; her abdomen is perceptibly leſs; and in other reſpects ſhe is much the ſame as at my laſt viſit.

Oct. 11th. The vomiting had recurred on the 7th, with greater violence than before, and had continued for the ſpace of nearly forty-eight hours. When ſhe was firſt ſeized with vomiting, ſome anti-emetic medicine might have been tried; and although I have very frequently seen vomiting produced by the system being incautiously overcharged with *Digitalis*, yet as I never witnessed any attendant phænomena which excited in me any considerable degree of alarm, I was not particularly ambitious to obviate this effect. Dr. WITHERING has mentioned as antidotes, *brandy and laudanum*; but he seems to place his chief dependence on the application of a *blister* to the region of the stomach. My patient's pulse is now reduced to *thirty-eight strokes of the artery in a minute*, and though tolerably full, is extremely irregular; the tumefaction of her abdomen is nearly gone; every other morbid symptom is evidently on the decline; she appears now to be approaching to a state of convalescence. Her pills are to be repeated, and she is to take only two of them daily.

I did not see her again till the 26th, when I found her very much improved. She told me, that the vomiting had again returned; and though not with the same violence as it had done before, yet that it had continued to recur occasionally for a week. I had previously remarked, that every attack of vomiting had been followed by beneficial effects; and it was apparent that this last had been succeeded by a very perfect state of convalescence. Her appetite is good; she is daily improving in strength; her pulse is 50. She expressed some fear of a relapse, and she purposes to take a pill or two every day till she is completely restored. About a month afterwards I saw her in health, and following her ordinary employment: I am informed, however, that about the middle of March last she again became indisposed, and again had recourse to her pills. What symptoms she laboured under, I know not; but from the circumstance of her having again applied to her former medicine, it is probable that she thought her complaints similar to those with which she had formerly been afflicted. How long she took the *Digitalis* a second time, and what its effects were, I have never heard; I am informed, however, that she was prescribed to by another physician. She is since dead.

CASE XIII.—Jan. 17, 1800. E. B. æt. 18, was seized on the 14th with violent rigors and other symptoms of pyrexia. She now complains of an acute pain in her left side; a hard, dry cough; great difficulty of breathing; and excessive thirst. If she attempts to lie down on either side, her cough is much aggravated, and she instantly experiences a sense of suffocation. She is supported by pillows, in a posture almost erect. Her tongue is much furred; her urine is high coloured; her bowels are tardy; her pulse is 110. She has been bled in the arm, and she has taken saline medicines from her medical attendant. I advised advised a dozen *leeches* to be applied to the region of the *thorax*, and the greatest number to be applied in the vicinity of that part of her side where the pain was so acute. I next ordered her *chest* to be covered with a very large *blister*: I then directed her to live on *toast and water* exclusively, but to drink even that sparingly; and I prescribed for her a grain of the *Digitalis*, with a small portion of the *Pulv. e Tragacanth. C.* to be taken in a solution of *Sal. Nitri* every three hours.

Jan. 18th. The blood discharged by means of the *leeches* was very considerable, without affording her much apparent benefit. The *blister* has operated well, without materially relieving the difficulty of breathing, which is now uncommonly distressing; her thirst is extreme; her cough is incessant, and is accompanied by a dark-coloured, greenish expectoration; her tongue is loaded with a thick, brown fur; her countenance is marked by a singular anxiety, and there is an apparent tendency to delirium; her pulse is 120, with a slight intermission. She has just had an alvine evacuation. I advised a continuance of her medicines and regimen.

Jan. 19th. Last evening, towards the approach of midnight, she experienced an aggravation of all her symptoms; and to those already enumerated was superadded a very harrassing *singultus*. After passing a most dreadful night, she sunk into repose about four or five o'clock this morning; she continued in that state for several hours, and awoke refreshed. She has had no considerable evacuation by the kidnies, the bowels, or skin; and yet there is a very evident abatement of every morbid symptom. Her difficulty of breathing is much relieved; her cough is less teazing, more free, and the matter expectorated looks better than before; her countenance is more natural; her tongue is much more clean. She can now lie down in a situation approaching to a horizontal one, and can, without much uneasiness, turn a little to either side. Her pulse is reduced to 96 strokes in the minute.

Jan. 20th. She has had an easy night, and again she has enjoyed some refreshing repose in the morning; she breathes with much ease, and she can, without much inconvenience, lie down on either side; her cough continues to be more relieved, and she expectorates freely a mucus of a laudable colour; her urine is more pale, and deposits a sediment; her pulse is 65. I enjoined the use of her medicine three or four times a day for a few days longer, as also her low regimen; I then took my leave of her. I saw this girl about a fortnight afterwards in the most complete health.

Case

Case XIV.—Feb. 4, 1800. G. L. æt. 35, on the evening of this date was ſeized with rigors, and other febrile ſymptoms. A reſpectable practitioner was conſulted, who immediately directed him to have an *emetic.* This draught operated well, but ſoon after its operation the patient expectorated a little blood. His pulſe was 120.

Feb. 5th. This morning he took an aperient draught with the *Pulv. Prophylact.* which was followed by powders compoſed of *Sal. Nitri*, *Pulv. Antimonial. Lond.* and *Pulv. e Tragacanth. C.* One of thoſe powders was directed to be taken in a proper vehicle every two or three hours. His pulſe and other ſymptoms were as before.

Feb. 6th. He had taken ſix of the powders above preſcribed. He complained of an acute pain in his left ſide, attended by an extreme difficulty of breathing, diſtreſſing cough, and bloody expectoration. He had a *bliſter* applied to his cheſt, and was ordered four grains of the *Digitalis* in ſix powders, to be conſumed in twenty-four hours.

Feb. 7th. He had taken his powders with the *Digitalis.* His pain was relieved by the *bliſter.* His breathing was ſtill laborious, and the hæmoptyſis continued to recur as before. The powders were repeated.

Feb. 8th. I ſaw this patient for the firſt time this morning, accompanied by his medical attendant. He laboured under a well-marked caſe of *pneumonic inflammation.* In addition to the common phænomena which characteriſe that diſeaſe in its moſt alarming form, I had reaſon to apprehend a ſupervention of delirium. A ſingular rapidity marked his looks and actions. His pulſe was 120; his reſpiration was laborious; his thirſt was extreme; he continued to expectorate pure arterial blood. I directed him to drink frequently of cold water; I repeated his powders, with an additional quantity of the *Pulv. Antimonial. Lond.* to be taken in a ſolution of *Sal. Nitri.*

Feb. 9th. He had paſſed a much eaſier night. The pain of his ſide had abated conſiderably; and the difficulty of breathing was much relieved. The expectoration of blood, however, did not appear to be leſſened in the ſmalleſt degree, though his pulſe was reduced ſo low as 70 ſtrokes in a minute. His medicines were repeated.

Feb. 10th. This morning exhibited a phœnomenon which ſtruck us with aſtoniſhment. His pulſe was full, regular, and *beat only forty strokes in the minute.* Every morbid ſymptom had begun then to diſappear; and the hæmorrhage from his lungs was now much leſſened, though he ſtill continued occaſionally to diſcharge blood. The medicines were repeated.

Feb. 11th. He appeared in a ſtate of convaleſcence. His pulſe pulse was still *forty*. We discontinued his powders; and he began to take a julep composed of *Conserv. Rosar. &c.* acidulated with the *Acid. Vitriolic. Dilut.* He took this julep till the 16th, at which period all his complaints had ceased. His pulse, however, still continued at *forty*; and it was not till some time afterwards that it recovered its usual velocity. This patient has ever since enjoyed the most perfect health.

CASE XV.—June 27th, 1800, B. C. ætat. 50, was suddenly seized with violently marked symptoms of *pneumonia*. An intelligent practitioner in the neighbourhood was consulted for him, but what medicines he gave him I know not. On the evening of July 1, I visited him, and found him labouring under symptoms very similar to those described in Case 13. I pursued a similar practice, and with equal success; a detail of the minutiæ is therefore unnecessary: but I ought to observe, that in this case the morbid symptoms were combated with much difficulty; the issue of the disease hung in suspense for upwards of a week; *and during the whole of that period he took daily from eight to twelve grains of the Digitalis in its most active state.* I visited him every morning, and with the most anxious impatience I expected the usual operation of the plant. It at last exerted its influence over him, and all his complaints disappeared. A few days ago I saw him in health.

CASE XVI.—August 4, 1800, J. B. æt. 35, was suddenly seized with symptoms of *pneumonic inflammation*. The whole region of the thorax was attacked by violent shooting pains; his thirst was great; the difficulty of respiration was extreme; his pulse beat 100 strokes in the minute. His apothecary took from him twelve ounces of blood, which exhibited a thick sizy surface. During that day he took powders composed of *Sal. Nitri* et *Pulv. Antim. Lond.* and in the evening a grain of *Digitalis* was added to each powder, and they were directed to be taken every three hours. At this period he complained of a most tormenting pain in his left side, which was covered with a *blister*.

Aug. 5th. This morning every morbid symptom was apparently increased. The pain of his side, he said, was much abated; but he now experienced the most excruciating sensation immediately under the *sternum*. The same quantity of blood was taken as before, and the same medicines were continued.

Aug. 6th. There was still an aggravation of the morbid symptoms. His pulse was 110; his thirst was extreme; his tongue was covered with a dark coloured fur; the difficulty of of reſpiration was great; his urine was remarkably high co-loured; he laboured under the conſtant recurrence of a bilious diarrhœa. Blood was again taken from his arm.

Aug. 7th. The operation of venæſection was once more performed. I ſaw him, for the firſt time, this morning. His pulſe was irregular, and beat 120 ſtrokes in the minute; he laboured extremely in his breathing. He was laid upon his back, ſupported by pillows, nor could he for a moment bear any other ſituation. His cough was hard, hollow, inceſſant; and when he expectorated any thing, it reſembled a dark green or brown ſerum. His countenance appeared ſunk, and indicative of approaching diſſolution. He faultered in his ſpeech, and there was a ſupervention of a ſlight delirium. I covered the whole ſurface of the *thorax* with a *bliſter*, and I preſcribed the following formula:

℞. Fol. Digital. *recenter pulverizat.* ℈ſs. Opii puri pulv. gr. iij. Pulv. Antimonial. Lond. ʒſs. Sal. Nitri ℥j. tere et divide in chartulas xii.—Sumat j. quaque 2nd. hora in cyatho aquæ gelidæ.

Aug. 8th. Appearances this morning were rather more fa-vourable. His pulſe was reduced to 110, and was more re-gular; nor did he experience the ſame lancinating pains in his cheſt. His *bliſter* had excoriated the whole ſurface of the *tho-rax*; and had induced a moſt violent *ſtrangury*, which was relieved by the exhibition of *mucilages.* He had taken his me-dicines with great regularity; and the cool regimen, enjoined at the beginning, was ſcrupulouſly obſerved.

Aug. 9th. He had paſſed a better night than uſual. His pulſe this morning was reduced ſo low as 96. He had not had ſo many bilious evacuations; but there was an appearance of yellowneſs in the *tunica albuginea* of his eyes; and his tongue began to be covered with a yellow cruſt. His reſpiration was certainly more eaſy; his cough was neither ſo conſtant, nor ſo painful; and the matter expectorated had aſſumed a more laudable colour and conſiſtence. A violent *ſingultus* had at-tacked him in the night, and ſtill continued to harraſs him extremely. I directed him to have the following medicines:

℞. Calomel. Fol. Digital. *recenter pulverizat.* a. gr. ſs. Pulv. Antimonial. Lond. gr. iij. Conſerv. q. s. ut fiat bolus, quaque 2nd. hora ſumendus cum Decoct. Nitros. ℥j. I alſo adviſed an *anodyne* at bed time.

Aug. 10th. He had paſſed an eaſier night. The *hiccup* was not ſo violent, nor did it recur ſo frequently as yeſterday; his pulſe was reduced to 84 ſtrokes in a minute; his tongue was more clean and moiſt. He expreſſed a deſire to have a little food. He was now obviouſly under the influence of the *Digi-*

*talis.*

*talis.* Indeed, I am inclined to afcribe to its operation the occurrence of *singultus.* This laft fymptom continued occafionally, for feveral days, to harrafs him, but was repeatedly, and very effectually obviated by the exhibition of *Sp. Nitri Dulc.* His pulfe began, from this period, to be reduced in point of frequency, till it approached its ufual ftandard. Every morbid fymptom difappeared, and he was reftored to the moft complete health.

[ To be continued. ]

